# Supplementary material for: Allosteric regulation and crystallographic fragment screening of SARS-CoV-2 NSP15 endoribonuclease
Source: Nucleic Acids Res. 2023 Apr 28;51(10):5255–70. doi: 10.1093/nar/gkad314 (PMC10250223; doi:10.1093/nar/gkad314)
Supplement: gkad314_Supplemental_Files [file gkad314_supplemental_files.zip › Supplementary datasets.zip!/139/Refine_1/01-BUSTER/Cycle-2/shell.01/R_Rfree.html]

# Rxpct factors in resolution bins at cycle 0


|  |  |  |  |  |  |  |  |  |
| --- | --- | --- | --- | --- | --- | --- | --- | --- |
|  | **<d> (Å)**  Rxpct\_Work | Rxpct\_Free | Rxpct\_All | |  |  |  |  |
|  | 13.2733 | 0.3222 | 0.4730 | 0.3286 |  |
|  | 7.6634 | 0.2648 | 0.2709 | 0.2652 |  |
|  | 5.9360 | 0.2671 | 0.2503 | 0.2664 |  |
|  | 5.0168 | 0.2030 | 0.2088 | 0.2033 |  |
|  | 4.4244 | 0.1755 | 0.1782 | 0.1757 |  |
|  | 4.0021 | 0.1674 | 0.1602 | 0.1671 |  |
|  | 3.6814 | 0.1845 | 0.2160 | 0.1860 |  |
|  | 3.4272 | 0.1847 | 0.1751 | 0.1842 |  |
|  | 3.2193 | 0.1750 | 0.1938 | 0.1760 |  |
|  | 3.0451 | 0.1730 | 0.1805 | 0.1734 |  |
|  | 2.8965 | 0.1823 | 0.1929 | 0.1828 |  |
|  | 2.7677 | 0.1775 | 0.1820 | 0.1777 |  |
|  | 2.6547 | 0.1836 | 0.2026 | 0.1848 |  |
|  | 2.5545 | 0.1888 | 0.1961 | 0.1892 |  |
|  | 2.4648 | 0.1887 | 0.1972 | 0.1891 |  |
|  | 2.3840 | 0.2010 | 0.2183 | 0.2018 |  |
|  | 2.3106 | 0.2001 | 0.1870 | 0.1995 |  |
|  | 2.2436 | 0.2088 | 0.2005 | 0.2083 |  |
|  | 2.1821 | 0.1975 | 0.1980 | 0.1976 |  |
|  | 2.1254 | 0.2066 | 0.1960 | 0.2061 |  |
|  | **Overall** | 0.1997 | 0.2094 | 0.2002 |  |

# Rxpct factors in resolution bins at cycle 1


|  |  |  |  |  |  |  |  |  |
| --- | --- | --- | --- | --- | --- | --- | --- | --- |
|  | **<d> (Å)**  Rxpct\_Work | Rxpct\_Free | Rxpct\_All | |  |  |  |  |
|  | 13.2733 | 0.3227 | 0.4736 | 0.3291 |  |
|  | 7.6634 | 0.2661 | 0.2721 | 0.2665 |  |
|  | 5.9360 | 0.2683 | 0.2520 | 0.2677 |  |
|  | 5.0168 | 0.2042 | 0.2099 | 0.2045 |  |
|  | 4.4244 | 0.1762 | 0.1777 | 0.1763 |  |
|  | 4.0021 | 0.1683 | 0.1596 | 0.1679 |  |
|  | 3.6814 | 0.1853 | 0.2159 | 0.1868 |  |
|  | 3.4272 | 0.1859 | 0.1758 | 0.1853 |  |
|  | 3.2193 | 0.1777 | 0.1956 | 0.1786 |  |
|  | 3.0451 | 0.1769 | 0.1817 | 0.1772 |  |
|  | 2.8965 | 0.1860 | 0.1945 | 0.1864 |  |
|  | 2.7677 | 0.1824 | 0.1860 | 0.1826 |  |
|  | 2.6547 | 0.1881 | 0.2099 | 0.1894 |  |
|  | 2.5545 | 0.1911 | 0.1995 | 0.1916 |  |
|  | 2.4648 | 0.1910 | 0.2002 | 0.1914 |  |
|  | 2.3840 | 0.2014 | 0.2226 | 0.2024 |  |
|  | 2.3106 | 0.1989 | 0.1833 | 0.1982 |  |
|  | 2.2436 | 0.2075 | 0.2024 | 0.2073 |  |
|  | 2.1821 | 0.1965 | 0.1970 | 0.1965 |  |
|  | 2.1254 | 0.2051 | 0.1959 | 0.2046 |  |
|  | **Overall** | 0.2012 | 0.2106 | 0.2017 |  |

# Rxpct factors in resolution bins at cycle 2


|  |  |  |  |  |  |  |  |  |
| --- | --- | --- | --- | --- | --- | --- | --- | --- |
|  | **<d> (Å)**  Rxpct\_Work | Rxpct\_Free | Rxpct\_All | |  |  |  |  |
|  | 13.2733 | 0.3233 | 0.4751 | 0.3297 |  |
|  | 7.6634 | 0.2668 | 0.2725 | 0.2672 |  |
|  | 5.9360 | 0.2694 | 0.2537 | 0.2688 |  |
|  | 5.0168 | 0.2054 | 0.2104 | 0.2057 |  |
|  | 4.4244 | 0.1770 | 0.1773 | 0.1771 |  |
|  | 4.0021 | 0.1692 | 0.1603 | 0.1688 |  |
|  | 3.6814 | 0.1859 | 0.2161 | 0.1873 |  |
|  | 3.4272 | 0.1866 | 0.1767 | 0.1860 |  |
|  | 3.2193 | 0.1789 | 0.1957 | 0.1798 |  |
|  | 3.0451 | 0.1782 | 0.1845 | 0.1785 |  |
|  | 2.8965 | 0.1871 | 0.1947 | 0.1875 |  |
|  | 2.7677 | 0.1835 | 0.1862 | 0.1836 |  |
|  | 2.6547 | 0.1893 | 0.2113 | 0.1907 |  |
|  | 2.5545 | 0.1918 | 0.2014 | 0.1923 |  |
|  | 2.4648 | 0.1909 | 0.2005 | 0.1914 |  |
|  | 2.3840 | 0.2010 | 0.2232 | 0.2020 |  |
|  | 2.3106 | 0.1984 | 0.1818 | 0.1977 |  |
|  | 2.2436 | 0.2071 | 0.2029 | 0.2069 |  |
|  | 2.1821 | 0.1960 | 0.1956 | 0.1960 |  |
|  | 2.1254 | 0.2040 | 0.1952 | 0.2036 |  |
|  | **Overall** | 0.2019 | 0.2112 | 0.2024 |  |

# Rxpct factors in resolution bins at cycle 3


|  |  |  |  |  |  |  |  |  |
| --- | --- | --- | --- | --- | --- | --- | --- | --- |
|  | **<d> (Å)**  Rxpct\_Work | Rxpct\_Free | Rxpct\_All | |  |  |  |  |
|  | 13.2733 | 0.3153 | 0.4715 | 0.3219 |  |
|  | 7.6634 | 0.2645 | 0.2536 | 0.2638 |  |
|  | 5.9360 | 0.2886 | 0.3207 | 0.2898 |  |
|  | 5.0168 | 0.2299 | 0.2422 | 0.2307 |  |
|  | 4.4244 | 0.2003 | 0.2260 | 0.2016 |  |
|  | 4.0021 | 0.1958 | 0.1934 | 0.1956 |  |
|  | 3.6814 | 0.2033 | 0.2492 | 0.2055 |  |
|  | 3.4272 | 0.2059 | 0.2138 | 0.2063 |  |
|  | 3.2193 | 0.1997 | 0.2246 | 0.2011 |  |
|  | 3.0451 | 0.1999 | 0.2092 | 0.2003 |  |
|  | 2.8965 | 0.2053 | 0.2191 | 0.2059 |  |
|  | 2.7677 | 0.2016 | 0.2181 | 0.2023 |  |
|  | 2.6547 | 0.2078 | 0.2299 | 0.2091 |  |
|  | 2.5545 | 0.2001 | 0.2262 | 0.2016 |  |
|  | 2.4648 | 0.1976 | 0.2188 | 0.1986 |  |
|  | 2.3840 | 0.1976 | 0.2294 | 0.1991 |  |
|  | 2.3106 | 0.1921 | 0.1866 | 0.1918 |  |
|  | 2.2436 | 0.1937 | 0.2060 | 0.1943 |  |
|  | 2.1821 | 0.1861 | 0.1760 | 0.1856 |  |
|  | 2.1254 | 0.1914 | 0.1890 | 0.1913 |  |
|  | **Overall** | 0.2160 | 0.2362 | 0.2170 |  |
